# Femoral parallelism: evaluation and impact of variation on canine hip dysplasia assessment

**DOI:** 10.3389/fvets.2023.1160200

**Published:** 2023-05-05

**Authors:** Pedro Franco-Gonçalo, Sofia Alves-Pimenta, Lio Gonçalves, Bruno Colaço, Pedro Leite, Alexandrine Ribeiro, Manuel Ferreira, Fintan McEvoy, Mário Ginja

**Affiliations:** ^1^Department of Veterinary Science, University of Trás-os-Montes and Alto Douro (UTAD), Vila Real, Portugal; ^2^Veterinary and Animal Research Centre (CECAV), UTAD, Vila Real, Portugal; ^3^Associate Laboratory for Animal and Veterinary Sciences (AL4AnimalS), Vila Real, Portugal; ^4^Department of Animal Science, UTAD, Vila Real, Portugal; ^5^Department of Engineering, UTAD, Vila Real, Portugal; ^6^Institute for Systems and Computer Engineering (INESC-TEC), Technology and Science, Porto, Portugal; ^7^Neadvance Machine Vision SA, Braga, Portugal; ^8^Department of Veterinary Clinical Sciences, Faculty of Health and Medical Sciences, University of Copenhagen, Copenhagen, Denmark

**Keywords:** dog, femoral parallelism, Norberg angle, hip congruency index, hip dysplasia

## Abstract

Adequate radiographic positioning on the X-ray table is paramount for canine hip dysplasia (HD) screening. The aims of this study were to evaluate femoral parallelism on normal ventrodorsal hip extended (VDHE) view and the effect of femoral angulation (FA) on Norberg Angle (NA) and Hip Congruency Index (HCI). The femoral parallelism was evaluated comparing the alignment of the long femoral axis with the long body axis in normal VDHE views and the effect of FA on NA and HCI on repeated VDHE views with different levels of FA. The femoral long axis in normal VDHE views showed a ranged of FA from −4.85° to 5.85°, mean ± standard deviation (SD) of −0.06 ± 2.41°, 95% CI [−4.88, 4.76°]. In the paired views, the mean ± SD femur adduction of 3.69 ± 1.96° led to a statistically significant decrease NA, and HCI, and femur abduction of 2.89 ± 2.12 led to a statistically significant increase in NA and HCI (*p* < 0.05). The FA differences were also significantly correlated with both NA differences (*r* = 0.83) and HCI differences (*r* = 0.44) (*p* < 0.001). This work describes a methodology that allows evaluation of femoral parallelism in VDHE views and the results suggest that femur abduction yielded more desirable NA and HCI values and adduction impaired NA and HCI values. The positive linear association of FA with NA and HCI allows the use of regression equations to create corrections, to reduce the influence of poor femoral parallelism in the HD scoring.

## 1. Introduction

Canine hip dysplasia (HD) is the most prevalent orthopedic disease in dogs worldwide. Although HD is an inherited disease, there is currently no reliable diagnostic genetic test available. Therefore, HD screening still depends on phenotypic assessment, namely by radiography, which for more than 50 years has remained the leading HD diagnostic imaging technique and the only recommended tool available to assist in selective breeding. This strategy prevents the breeding of affected dogs and genetic disease transmission to offspring (1, 2).

Given the global importance of HD, there are several radiographic scoring systems in the world, the *Fédération Cynologique Internationale* (FCI) system is used in most European countries, the British Veterinary Association/Kennel Club system in the United Kingdom and the Orthopedic Foundation for Animals system is used in the United States of America (3, 4). Despite the diversity, some evaluation parameters play a common role in some of these classification systems. In particular, the Norberg Angle (NA) and the visual assessment of hip joint congruency are important metrics to evaluate hip joint conformation (4, 5). These parameters are crucial for distinguishing HD categories, especially, for differentiating between joints with no evidence of HD, near-normal hip joints, and mild HD (6). A recent study proposes an objective computer-assisted methodology, the Hip Congruency Index (HCI), to assess congruency in the form of a numerical value, decreasing the subjectivity linked to the human scrutineer when assessing this essential parameter (7).

Guidelines to ensure the technical quality of radiographs in the ventrodorsal hip extended (VDHE) view were defined in the 1960s, and are still recommended today by worldwide scoring organizations for certification purposes (2). According to the standard radiographic technique, it is required that the dog is positioned in dorsal recumbence with the hind limbs extended caudally and the femurs parallel to the spine, to the top of the table, and to each other, while the patellae are centered over the femoral shafts (3).

It is already recognized that inappropriate positioning on the X-ray table results in poor radiographical technical quality since it is associated with alteration of the projected anatomical relationship between the femoral head and the acetabulum (8). To some extent, this leads to misinterpretation in HD scoring by the scrutineers, which inevitably precipitates incorrect selection of dogs for breeding (9).

Previous studies in cadavers have already evaluated the level of pelvic tilting along the long axis of the body, as well as femoral supination and pronation, and their influence on the NA and hip joint congruency in VDHE views (10, 11). One of these studies concluded that femoral pronation benefits the relationship between the femur (false negative) and acetabulum while supination impairs it (false positive) using in evaluation the NA (11). However, other clinical study showed that the NA measurement was not affected significantly by these conditions (12). Regarding pelvic tilting, clinical studies have shown similar results to studies on cadavers, in which the appearance of optimal coxofemoral joint relationship, characterized by the NA is favored in the upperside of the rotation and impaired in the underside (12). Also, another study has reported that pelvic tilting along the short axis of the pelvis did not affect the NA measurement (13).

Although different recommended technical quality positioning parameters have been thoroughly studied, the parallelism of the femurs, to the authors` knowledge, has never been quantified in VDHE views. There are currently no studies that propose an objective method for measuring femoral parallelism or on its impact on HD classification. Thus, this assessment remains somewhat subjective when accepting or rejecting a hip radiograph for HD scoring (14, 15).

Therefore, the main objectives of this study were to propose a method to measure the femoral parallelism relative to the long axis of the body and to investigate whether the variation in this parameter has a significant impact on the metrics used for HD scoring such as NA and the HCI. Our null hypothesis is that femoral adduction and abduction does not interfere in these HD scoring parameters.

## 2. Materials and methods

This was a retrospective observational study based on the evaluation of VDHE radiographs performed between 2010 and 2022 for HD screening. The radiographs were selected from the Veterinary Teaching Hospital of the University of Trás-os-Montes and Alto Douro and the Danish Kennel Club databases. Recorded data included breed, sex and age. This study was subdivided in two different parts. First, 50 VDHE radiographs (100 hips), which were considered by one of the authors (MG) to have adequate positioning and normal technical quality for HD scoring, of 50 dogs were randomly selected from the database. These radiographs were used to assess and define the normal parallelism between the femurs and the long axis of the body. In the second part, 126 VDHE radiographs (252 hips) of 63 dogs were used. Each of dogs had two repeated VDHE radiographs obtained on the same day, one with at least one of the femurs positioned with better parallelism to the long axis of the body and another radiographs (poorly positioned) showing the same femur as the first but with a greater degree of femoral adduction or abduction.

The inclusion criteria were dogs older than 12 months of age, with no severe signs of HD, in which radiographic reference landmarks were clearly visible. All radiographs were required to have the patellae superimposed over the femoral condyles with medial or lateral patellar indices ≥0.45, and maximum pelvic tilting along the long axis of the body of 2 degrees (11, 16). In duplicate (poorly positioned) radiographs, the femoral axis was required to have an angulation (degree of adduction or abduction) relative to the long axis of the body greater than 1 degree compared to its normal pair. Due to the observational nature of the study, the ethical committee approval and the owner’s consent were waived.

### 2.1. Radiographic measurements

All images were in DICOM format and the measurements were performed by the same examiner (PG) using an image analysis software (Dys4Vet version 2.0, accessed between June and December 2022).

**Figure 1:**
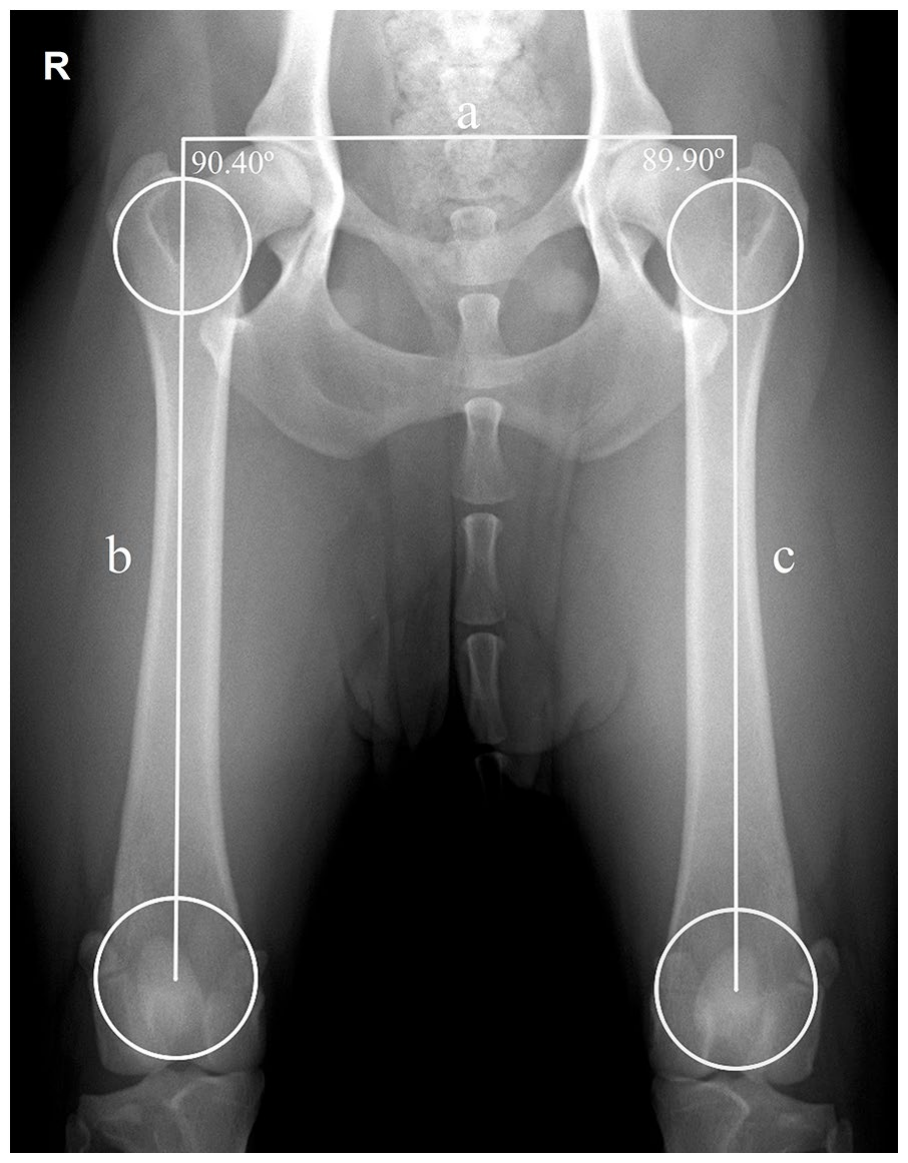
Assessment of the long axis of the femur using Symax-based method in a normal ventrodorsal hip extended view with adequate dog positioning. A line was drawn between the center of the proximal and distal femoral circles. The proximal and distal femoral circles were drawn touching 3 points each, the proximal circle touches the medial and lateral cortical bone and the distoproximal boundary of the femoral neck; the distal circle touches the medial and lateral cortical bone and the boundary of the intercondylar groove. Application of the parallelism evaluation method, illustrating the transverse pelvic axis (a) that joins the right and left craniolateral acetabular borders, and connects the right long femoral axis (b) with the left long femoral axis (c). The right femur shows a slight abduction of 0.40° (90.40° − 90° = 0.40°) and the left femur a slight adduction of −0.10° (89.90° − 90° = −0.10°). R: right side.

The long axis of the femur was defined using a Symax-based method (17) (Figure 1). The long axis of the femur was determined by drawing a straight line joining the centers of two circles: one circle drawn on the proximal femoral shaft and another on the distal shaft. The boundaries of the proximal and distal femoral circles touch three points, one on the proximal boundary of the femoral neck or intercondylar groove, respectively; and the other two on the medial and lateral metaphyseal cortical bone.

**Figure 2:**
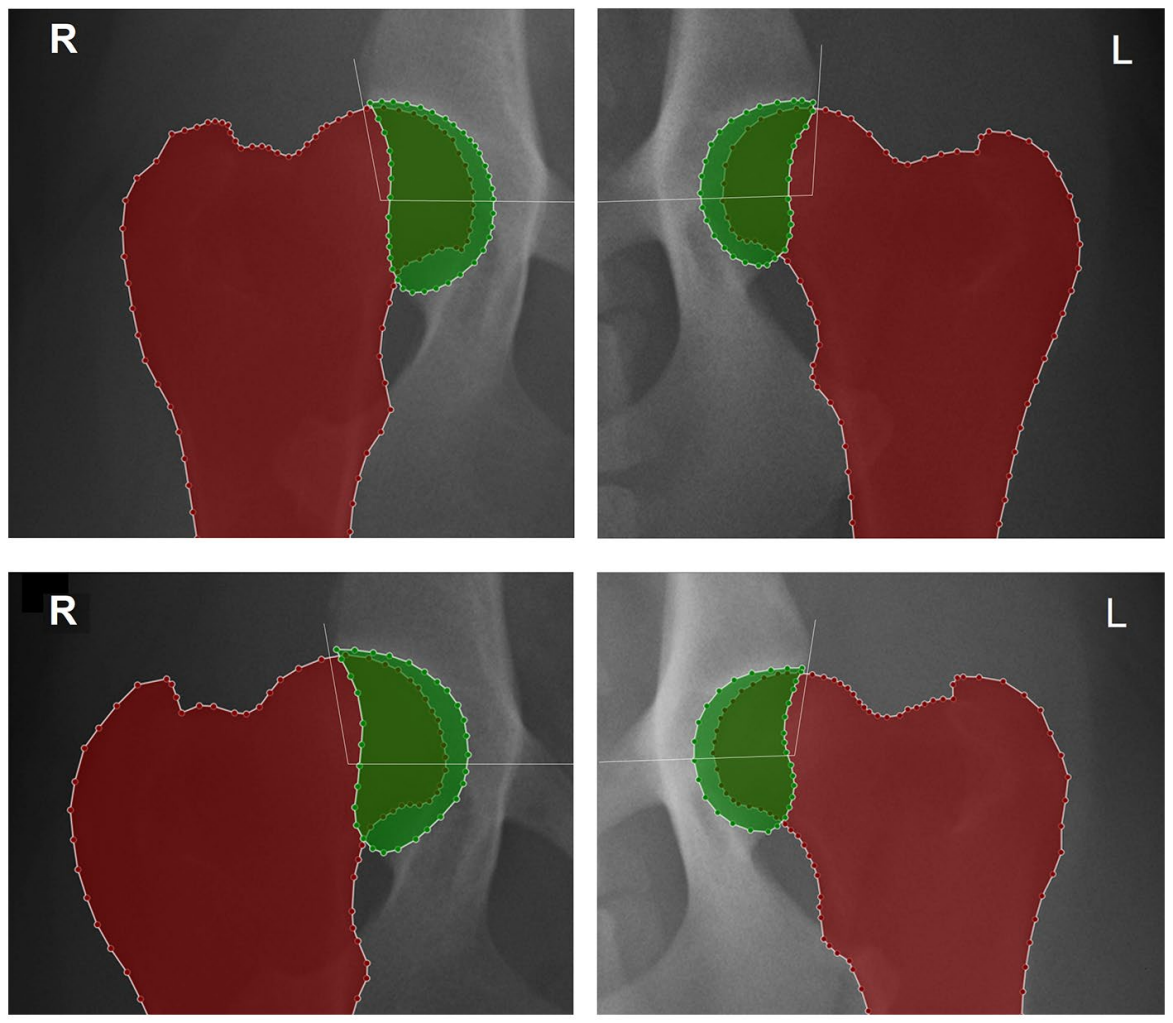
Detail of the right (R) and left (L) hip joints of two different dogs and delimitation of the proximal femur and acetabulum using LabelMe annotation tool to calculate the hip congruency index: the acetabular area is colored in green, the proximal femur in red and acetabular area occupied by the femoral head is showed by the overlap between the colorful areas. The white lines represent the hip Norberg angle. On top femur positioning is considered with normal femoral parallelism showing a hip congruency index and Norberg angle, respectively: right side 0.72 and 101.0°; left side 0.65 and 95.3°. At the bottom, repeated views showing a femoral angulation, a hip congruency index and Norberg angle, respectively: right side adduction of 7.5°, 0.65 and 99.6°; left side abduction of 5.8°, 0.68 and 100.6°.

The long axis of the body was considered perpendicular to the transverse pelvic axis defined, as a line joining the two craniolateral acetabular edges. The angulation of each femur (FA) relative to the long axis of the body was determined by measuring the interior angle between the lines drawn to define the transverse pelvic axis and the long femoral axis. The deviation of angulation was calculated by subtracting 90 degrees from the measured angle between transverse pelvic axis and the long axis of the femur (Figure 2). A positive or negative angulation deviation was considered as femoral abduction or adduction, respectively.

In the second part of the study, the femoral angulation was determined for each femur, and the NA and HCI were measured for each hip, in all pairs of radiographs.

The NA was calculated between the line joining the centers of the femoral heads and another line connecting the center of the femoral head to the ipsilateral craniodorsal effective acetabular rim (1, 18). The HCI was calculated by image segmentation delineating the acetabulum and the femur and dividing the acetabular area occupied by the femoral head by the acetabular area (7, 19).

### 2.2. Statistical analysis

Statistical analysis was performed using the computer software SPSS (SPSS Statistics for Windows Version 27.0: IBM Corp., Armonk, NY, United States).

The Central Limit Theorem was adopted, which entails that for sufficiently large sample sizes (*n* > 30) the distribution tends to be normally distributed, independently of the distribution of the population from where it originated and so parametric tests for data analysis were used (20). The data analysis was performed on femurs and joints individually. In the first part of the study, descriptive statistical analysis was used to detail the FA in normal VDHE views. In the second part, the pairs in the sample were divided into duplicate and adequate sets, and then subdivided in 2 subsets each. Subdivision of duplicate set into duplicate adduction (Ad) and duplicate abduction (Ab) subsets and adequate set subdivided into adequate Ad and adequate Ab subsets. This approach is intended to highlight the influence that femur long axis displacement had from adequate positioning to poor, in NA and HCI HD scoring parameters.

The paired t-test was used to compare FA on the right and left side of normal VDHE views in the first part of the study, and whether NA and HCI values differed significantly between adequate and the poorly femoral positioned sets in the second part (21). Pearson correlation analyses were used to determine the association between FA differences and both NA and HCI differences in the paired radiographs. Regression models (i.e., y = ax + b) were used to predict NA and HCI corrections (y), based on FA variations (x), in degrees (22). A *p*-value of <0.05 was considered statistically significant. In significant differences, Cohen’s *d*, or standardized mean difference, was used to measure the effect size: small ≥0.20, medium ≥0.50, and large ≥0.80 (23).

## 3. Results

The 113 dogs included in this study consisted of 11 different breeds: Portuguese Pointers (56/113, 50%), Estrela Mountain dogs (34/113, 30%), Transmontano Mastiffs (8/113, 7%), Bernese Mountain dogs (5/113, 4%), Labrador Retrievers (3/113, 3%), German Shorthaired Pointers (2/113, 2%), Doberman (1/113, 1%), German Shepherd (1/113, 1%), Gordon Setter (1/113, 1%), Hungarian Pointer (1/113, 1%), and Portuguese Water dog (1/113, 1%). There were 51 (45%) males and 62 (55%) females. The age ranged from 12 to 129 months, the mean ± standard deviation (SD) was 28.68 ± 21.94 months.

### 3.1. Parallelism between the femurs and the long axis of the body on normal VDHE views (first part)

The femoral long axis deviation relative to the long body axis in the VDHE normal views ranged from −4.85° to 5.85° with mean ± SD of −0.06 ± 2.41°, 95% Confidence Interval [−4.88, 4.76°]. In the right side was registered a mean FA deviation of 0.20 ± 2.29° and in the left side 0.08 ± 2.53° (*p* = 0.526, in paired t-test) (Table 1).

**Table 1:** Descriptive statistics and paired *t*-test of the parallelism between the long femoral axis and the long axis of the body.

	Axis	*n*	Mean ± SD (in degrees)	Range	*p*-value
Femoral Angulation	Right	50	−0.20 ± 2.29	−4.77 to 5.79	0.526
	Left	50	0.08 ± 2.53	−4.85 to 5.85		Deviation
	Both	100	−0.06 ± 2.41	−4.85 to 5.85	–

SD, standard deviation.

### 3.2. Effect of femoral adduction and abduction on the NA and the HCI (second part)

Of the 126 pairs of femurs only 80 pairs femurs met the predefined inclusion criteria, 38 femurs underwent adduction and 42 underwent abduction on the duplicate radiography set.

#### 3.2.1. Femoral adduction

In the adequate Ad subset the mean ± SD FA was 89.51 ± 1.90°, the NA was 100.88 ± 5.46° and the HCI was 0.66 ± 0.09; and in the duplicate Ad subset, the FA was 85.82 ± 2.11°, the NA was 99.04 ± 5.45° and the HCI was 0.65 ± 0.08°. The FA differences between adequate and duplicate subsets ranged from 1.06 to 9.09°, mean ± SD of 3.69 ± 1.96°. The differences on adduction subsets were statistically significant for both parameters (*p* < 0.05, in paired *t*-test). Both the NA and the HCI showed small effect sizes (*d*_NA_ = 0.34, *d*_HCI_ = 0.20) (Table 2).

**Table 2:** Norberg angle and hip congruence index on adequate and duplicate radiographs accordingly to femur adduction or abduction displacement.

Femur displacement	Subsets	*n* pairs	Femoral angulation (mean ± SD; in degrees)	Femoral angulation differences (mean ± SD and range, in degrees)	Norberg angle (mean ± SD; in degrees)	Hip congruency index (mean ± SD)
Adduction	Adequate Ad	38	89.51 ± 1.90^a^	3.69 ± 1.96, 1.06 to 9.09	100.88 ± 5.46^a^	0.66 ± 0.09^a^
	Duplicate Ad		85.82 ± 2.11^b^		99.04 ± 5.45^b^	0.65 ± 0.08^b^
Abduction	Adequate Ab	42	90.77 ± 2.29^a^	−2.89 ± 2.12, −9.43 to −1	100.57 ± 5.84^a^	0.66 ± 0.07^a^
	Duplicate Ab		93.65 ± 2.12^b^		102.10 ± 5.45^b^	0.67 ± 0.07^b^

Ad, adduction; Ab, abduction; SD, standard deviation.

Different superscripts, in femoral adduction and abduction groups and in each separate variable (femoral angulation, Norberg angle and hip congruence index) indicate statistically significant differences between subsets (*p* < 0.05).

#### 3.2.2. Femoral abduction

In the adequate Ab subset the mean ± SD FA was 90.77 ± 2.29°, the NA was 100.57 ± 5.84° and the HCI was 0.66 ± 0.07; and in the duplicate Ab subset, the FA was 93.65 ± 2.12°, the NA was 102.10 ± 5.45° and the HCI was 0.67 ± 0.07. The FA differences between subsets ranged from −9.43 to −1° with mean ± SD of −2.89 ± 2.12°. The differences on abduction subsets were statistically significant for both parameters (*p* < 0.05, in paired *t*-test). The NA showed a small effect size (*d*_NA_ = 0.27), whereas the HCI showed a negligible effect size (*d*_HCI_ = 0.15) (Table 2).

#### 3.2.3. Correlation between FA differences and HD scoring metrics differences

The FA differences were significantly correlated with NA differences (*r* = 0.83, 95% CI [0.75–0.89], *p* < 0.001). A significant regression equation was found (*R*^2^ = 0.68, *p* < 0.001). The regression model was: NA differences = −0.02 + 0.44 × FA differences.

The FA differences were significantly correlated with HCI differences (*r* = 0.44, 95% CI [0.24–0.60], *p* < 0.001). A significant regression equation was found (*R*^2^ = 0.19, *p* < 0.001). The regression model was: HCI differences = 0.001 + 0.004 × FA differences.

## 4. Discussion

The HD radiographic diagnosis still plays a determinant role in the selection of breeding stock in the dog. The correct positioning of the dog is essential for an adequate radiographic interpretation and HD scoring of a VDHE view. The NA and the femoral head coverage, HD scoring essential parameters, are influenced by dog positioning on the X-ray table (6, 8).

Although the criteria for correct positioning are quite clear, the decision to accept or refuse a radiograph is still subjective, based on the skill and experience of the scrutineer’s, who tries to consider the relationship between misposition and its influence on HD scoring parameters. This subjectivity leads to some inconsistency among individual scrutineers, which can be detrimental for breeding and genetic improvement of dog breeds (15).

We present a methodology to evaluate the parallelism of the femurs relative to the long axis of the body with the purpose of improving the reproducibility radiographic imaging analysis. As far as we know no objective investigation has been carried out on this topic. In this study, reference values for the deviation between −5° and +5° in femoral parallelism for normal VDHE views with adequate positioning quality are suggested. Few VDHE views are perfect and therefore some femoral angulation/deviation must be tolerated. When comparing the right and left femoral axis we noticed that the positioning technique performed by the veterinarian was consistent on both sides, since there were no significant differences between the FA deviations of both sides (*p* > 0.05). The femoral angulation deviations between −5 to +5° in normal views are considerable acceptable. However, any grade of FA could theoretically interfere with femoral head and acetabulum radiographic relationship. It would be expected that the femoral angulation of the right and left femur would be different and influenced by a right/left-handed radiologist veterinarian, as it would be assumed that he would inadvertently apply more force to his dominant hand. This methodology has a weak point, it has difficulties assessing femoral angulation in severe cases of HD. Remodeling of the craniolateral acetabular edge, place of the landmark points defining the transverse pelvic axis are potentially indistinct or asymmetric on both sides, ultimately skewing the long axis of the body. However, that aspect does not seem very relevant, because in severe cases of HD the NA and HCI are not determinant for the final HD scoring.

In the second part of this study, we rejected the null hypothesis, since femoral abduction favors and adduction impairs the NA and HCI. The NA and hip congruency assessment are HD scoring parameters used in many HD scoring systems around the world (4). Changes in the NA and hip congruency related with dog mispositioning in the X-ray table can be understood by biomechanical principles. Femoral adduction promotes separation of the femoral head from the acetabulum resulting in lower NA and HCI due to a decrease in acetabular coverage of the femoral head, while femoral abduction favors both parameters, promoting proximity of the femoral head and the acetabulum. We used Pearson correlation to study the strength of the relationship between FA and the HD scoring parameters. A strong positive correlation was found between FA differences and NA differences. The NA regression model predicted that for each additional degree of FA, there was an average increase of 0.42 degrees in the NA or the opposite, a reduction of FA results in a NA reduction of the same magnitude (*p* < 0.001). The *R*^2^ depicts that 68% of the variance of the NA is explained by the variance of the FA. A moderate positive correlation was found between FA differences and HCI differences. The HCI regression model predicted that for each additional degree of FA, there was an average increase of 0.005 in the HCI and a reduction of FA results in a similar HCI reduction (p < 0.001). The *R*^2^ depicts that 19% of the variance of the HCI is explained by the variance of the FA. These findings confirm the hypotheses that FA is positively or negatively associated with both NA and HCI (22). The linear association and the generated regression models have the potential to be useful assets to predict corrective adjustments in these parameters. The NA corrections can be particularly important in the transition from FCI categories A to B and B to C, in which the NA and HCI are very decisive parameter.

There are some intrinsic anatomical factors that affect hip joint motion: muscles as active constraints, the femoral head ligament as a passive constraint, and the geometry of osseous and cartilaginous structures (24). These factors vary greatly between animals and dog breeds (25). Some of these factors may influence the direction and range of motion of the femoral head when the hind limb moves in a radiographic evaluation in VDHE projection. Presumably, the lateral and medial pelvic muscle mass, as well as. The femoral head ligament, can modulate the translational motion of the femoral head when a hind limb undergoes adduction or abduction (24, 26). The translational lateral motion of the femoral head may not be as pronounced in muscular dogs, as it might be in dogs with less muscle mass (27). Furthermore, a dog with more developed muscle mass requires more strength from the veterinarian to achieve the desirable femoral parallelism, this usually causes a lesser degree of femoral pronation and adduction than is advisable, and as a result an unforeseen motion of the femoral head inside the hip joint, resulting in unexpected changes in the NA value and in hip joint congruency (11). We also advocate the idea that the extension of the hind limb and distal tension on the hip is greater as the femur is progressively adducted, subjecting the femoral head to a craniocaudal force vector, which causes it to follow a distal caudal trajectory. For the HD scoring parameters under study, the NA is most affected by this effect, because as the center of the femoral head moves away from the craniodorsal acetabular rim, moving in a caudolateral direction, the NA value decreases slightly. The HCI does not seem to be so affected, because the aforementioned motion of the femoral head does not result necessarily in a reduction in the acetabular area occupied by the femoral head. Furthermore, in our study, the NA demonstrated slightly larger effect sizes than the HCI. In canine HD, the morphological remodeling changes in both bone and cartilage are a response to mechanical stress on the hip joint (28). The HCI segmentation method used does not differentiate between healthy bone contour and bone remodeling or neoformation which inadvertently overestimates HCI values in dogs with coxofemoral osteoarthritis (7). Although we did not consider hips with severe dysplasia for the study, some cases presented mild signs of osteoarthritis. The alteration of the shape of the femoral neck is one of the primary signs of HD, and it tends to thicken with the progression of the disease (29). When the femur is adducted in a VDHE view some portion of the medial femoral neck lies within the projected acetabular coverage area, so a thick femoral neck would occupy more area over the acetabulum than a thin femoral neck of a healthy hip joint. Thus, this is an additional fact that explains some of the variability in our results.

Some of the limitations of this study are due to its retrospective and observational nature, we could not evaluate the variation of HD scoring metrics across a large predetermined range of FA degrees, which limited the scope of the study. Future prospective cadaveric studies or similar studies with a larger sample and different dog breeds can be helpful to better understand the effect of FA on HD assessment.

In conclusion, this study describes a methodology that allows evaluating femur parallelism in the VDHE view, that can be used in future with confidence as a criterion for rejecting radiographs for canine HD scoring. The femur abduction and adduction had significant impact on NA and HCI. Femur abduction favors NA and HCI and adduction impairs NA and HCI.

## Data availability statement

The raw data supporting the conclusions of this article will be made available by the authors, without undue reservation.

## Ethics statement

Ethical review and approval was not required for the animal study because due to the observational nature of the study (retrospective analysis of radiographic images of a database), the ethical committee approval and the owner’s consent were waived.

## Author contributions

MG and PF-G contributed to conception and design of the study. BC, FM, MF, PF-G, PL, SA-P, and MG organized the database. AR, MG, PF-G, and PL defined the methodology. BC, FM, LG, MF, and MG performed validation and data analysis. PF-G and MG wrote the first draft of the manuscript. All authors contributed to the article and approved the submitted version.

## Funding

This work was financed by project Dys4Vet (POCI-01-0247-FEDER-046914), co-financed by the European Regional Development Fund (ERDF) through COMPETE2020 - the Operational Programme for Competitiveness and Internationalization (OPCI).

## Conflict of interest

Authors AR, MF, and PL were employed by Neadvance Machine Vision SA.

The remaining authors declare that the research was conducted in the absence of any commercial or financial relationships that could be construed as a potential conflict of interest.

## Publisher’s note

All claims expressed in this article are solely those of the authors and do not necessarily represent those of their affiliated organizations, or those of the publisher, the editors and the reviewers. Any product that may be evaluated in this article, or claim that may be made by its manufacturer, is not guaranteed or endorsed by the publisher.
